# Genome sequence of the yeast *Candida solani* UCD1087, isolated from soil in Ireland

**DOI:** 10.1128/mra.01168-24

**Published:** 2025-03-28

**Authors:** Matthieu Osborne, Conor Hession, Shareen Behnass, Christina Biju, John Dennison, Adam Faulkner, Anthony Flynn, Joshua Haugh, Sebastian Magner, Sean O'Reilly, Reema Saoud, Tadhg Ó Cróinín, Kenneth H. Wolfe, Geraldine Butler, Kevin P. Byrne

**Affiliations:** 1 School of Medicine, Conway Institute, University College Dublin, Belfield, Ireland; 2 School of Biomolecular and Biomedical Science, Conway Institute, University College Dublin, Belfield, Ireland; University of Maryland School of Medicine, Baltimore, Maryland, USA

**Keywords:** yeasts, genome analysis

## Abstract

*Candida solani* is a member of the Wickerhamomyces clade of budding yeasts. We present the genome sequence of *C. solani* strain UCD1087, which was isolated from soil on the University College Dublin (UCD) campus in Ireland. This genome is 12.85 Mb and was assembled into six chromosome-sized contigs plus a mitochondrial genome contig.

## ANNOUNCEMENT

*Candida solani* is an asexual species that is a member of the Wickerhamomyces clade of budding yeasts (1). It was first isolated from a potato starch mill in the Netherlands (2). It has no known food or biotechnological applications. As it does not grow above 35°C, it is unlikely to have pathogenic capacity in humans. The only other genome assembly available for this species is from the type strain (NRRL Y-2224), which is fragmented into over 9,000 contigs (1).

*C. solani* UCD1087 was isolated from soil collected on the University College Dublin (UCD) campus, Ireland (GPS coordinates 53.3085254, –6.2220592) as part of an undergraduate research module (3).

Soil material was passaged twice at room temperature in 9 mL liquid yeast extract–peptone–dextrose (YPD) containing chloramphenicol (30 µg/mL) and ampicillin (100 µg/mL) and cultured on YPD agar plates. A single colony was isolated. The species was identified by PCR and Sanger sequencing of the ribosomal DNA internal transcribed spacer (ITS) and D1/D2 regions (accession numbers PQ438597 and PQ438670), using primers ITS1 (TCCGTAGGTGAACCTGCGG) and ITS4 (TCCTCCGCTTATTGATATGC) (4), and for the D1/D2 region, NL1 (GCATATCAATAAGCGGAGGAA) and NL4 (GGTCCGTGTTTCAAGACGG) (5). Sequence identity was 100% (554/554 bp) in the ITS and 98.94% (559/565 bp) in the D1/D2 region to the type strain of *C. solani* (accession numbers KY102402 and NG_055191).

DNA was isolated by phenol/chloroform extraction from liquid YPD cultures grown at 20°C. Short-read library construction (Illumina DNA-Prep(M) Ref:20060060) and sequencing (UCD Conway Core Facility, Dublin, Ireland) used a NextSeq2000 and P1 flowcell, yielding 5.6 million read pairs (2 × 150 bp). Adapters and low-quality reads were removed (Skewer v0.2.2) (6). For long-read sequencing, after DNA extraction (Biosearch Technology Masterpure yeast DNA purification kit MPY80010), library preparation (Native Barcoding Kit SQK-NBD112-24), end-repair (NEB-M6630), and ligation (NEB-E6056), we selected for DNA >3 kb with long fragment buffer before Oxford Nanopore sequencing (MinION MK1C, flowcell FLO-MIN112 R10.4). Default fast basecalling was done on the instrument (MinKNOW v23.07.12; default demultiplexing; barcode trimming; reads ≥500 bp kept; no quality cut-off). NanoFilt (v2.3.0) (7) retained 98,802 reads with quality ≥10 and length ≥10,000 bp (reads N50 = 18,304 bp), which were then assembled using Canu (v2.2) (8), followed by five rounds of error correction using NextPolish (v.1.4.1) with the Illumina reads (9). Default parameters were used, except where otherwise noted.

The assembly consists of six nuclear contigs (total 12.85 Mb; N50 = 2,132,486 bp) and the mitochondrial genome (37,835 bp circular unit; accession number CAXWWC010000007). All six nuclear contigs appear to be complete chromosomes because every end of every contig is either a probable telomere repeat sequence (GGGATGTACTGTGTGGTGTA)_n_ (10) or an rDNA array (detected by BLASTN) (Table 1). All rDNA arrays are oriented with the 18S and 26S rRNA genes transcribed towards the nearby contig end.

**TABLE 1:** Genome assembly characteristics of *Candida solani* UCD1087

Chromosome	Size	Left end	Right end
1	3,520,480	rDNA array	Telomere
2	2,242,305	rDNA array	rDNA array
3	2,132,486	Telomere	Telomere
4	1,914,830	Telomere	rDNA → Telomere
5	1,507,218	rDNA array	Telomere
6	1,498,922	Telomere	Telomere
Mitochondrial	37,835	n/a (circular)	n/a (circular)

The UCD1087 genome assembly has an average nucleotide identity (11) of 96.26% to the assembly of the *C. solani* type strain NRRL Y-2224 (accession no. JAKVQS010000000) (1). Using BUSCO v5.1.2 (12), genome completeness was estimated as 93.67% compared with the Ascomycota lineage data set. G + C content is 39.63%.

## Data Availability

This whole-genome shotgun project has been deposited as DDBJ/ENA/GenBank accession number CAXWWC010000000 (BioProject no. PRJEB80645). The version described in this paper is version 1. The reads were deposited at ENA (accessions ERR13731958, ERR13731962). The ITS sequence is PQ438597. The D1/D2 sequence is PQ438670. Isolate UCD1087 has been deposited in the CBS and PYCC culture collections as CBS 18653 and PYCC 10020.
